# Development of an Immunochromatographic Test for Diagnosis of Visceral Leishmaniasis Based on Detection of a Circulating Antigen

**DOI:** 10.1371/journal.pntd.0003902

**Published:** 2015-06-30

**Authors:** Chun-hua Gao, Yue-tao Yang, Feng Shi, Jun-yun Wang, Dietmar Steverding, Xia Wang

**Affiliations:** 1 National Institute of Parasitic Diseases, Chinese Center for Disease Control and Prevention, Laboratory of Parasite and Vector, Ministry of Public Health, National Centre for International Research on Tropical Diseases, WHO Collaborating Center for Malaria, Schistosomiasis and Filariasis, Shanghai, China; 2 BioMedical Research Centre, Norwich Medical School, Norwich Research Park, University of East Anglia, Norwich, United Kingdom; Royal Tropical Institute, NETHERLANDS

## Abstract

**Background:**

Visceral leishmaniasis (VL) is a life-threatening disease caused by protozoan parasites of the *Leishmania donovani* complex. Early case detection followed by adequate treatment is essential to the control of VL. However, the available diagnostic tests are either invasive and require considerable expertise (parasitological demonstration of the parasite in tissue smears) or unable to distinguish between past and active infection (serological methods). Therefore, we aimed to develop a lateral flow assay in the form of an immunochromatographic test (ICT) device based on the detection of a circulating *Leishmania* antigen using monoclonal antibodies (mAbs).

**Methodology/Principal Findings:**

mAbs were produced by fusion of murine myeloma cells with splenocytes isolated from a mouse immunized with *L*. *donovani* soluble crude antigen. Out of 12 cloned hybridoma cell lines, two secreted mAbs recognizing the same leishmanial protein. These mAbs were used to produce an ICT as a sandwich assay for the detection of circulating antigen in serum and blood samples. The ICT was evaluated with 213 serum samples from VL patients living in VL endemic areas in China, and with 156 serum samples from patients with other diseases as well as 78 serum samples from healthy donors. Sensitivity, specificity and diagnostic efficiency of the new ICT was 95.8%, 98.7% and 97.3%, respectively. Compared with a commercially available antibody detecting ICT, our antigen-based ICT performed slightly better.

**Conclusion/Significance:**

The newly developed ICT is an easy to use and more accurate diagnostic tool which fulfils the performance and operational characteristics required for VL case detection under field and laboratory conditions. As our ICT detects a circulating antigen, it will also be useful in monitoring treatment success and diagnosing VL in immunocompromised patients.

## Author Summary

Visceral leishmaniasis is a neglected disease caused by different species of protozoan parasites of the genus *Leishmania*. The disease is endemic in 61 countries, and in many of them it poses a serious public health issue. As visceral leishmaniasis is fatal if left untreated, early diagnosis is essential for treatment and control of the disease. Current available diagnostic tests are either difficult to carry out under field conditions or insufficiently accurate. In this study we developed a new diagnostic test which detects a circulation parasite-derived antigen in serum or blood samples of patients with visceral leishmaniasis. We found that our test performed better than most other tests based on the detection of parasite-specific antibodies. In addition, as our test is a device for the detection of an acute infection, it will be useful to validate treatment and in the diagnosis of the disease in patients with deficient antibody production (as in AIDS patients).

## Introduction

Visceral leishmaniasis (VL), or kala-azar, is a vector-borne disease caused by protozoan parasites belonging to the *Leishmania donovani* complex, which includes *L*. *donovani*, *L*. *infantum* and *L*. *chagasi*. The parasites infect and multiply preferentially in macrophages of the spleen, liver, bone marrow, and lymph nodes of their mammalian host. VL is a systemic disease and is fatal if left untreated. The disease is endemic in large areas of the tropics, subtropics and the Mediterranean Basin affecting 61 countries. It is estimated that approximately 200,000 to 400,000 cases of VL occur annually and that about 20,000 to 40,000 people die each year from the disease [1]. VL is also an important public health problem in China being endemic in six provinces or autonomous regions in western China including Xinjiang, Gansu, Sichuan, Shaanxi, Shanxi and Inner Mongolia [2–4]. The causative agents of VL in China are *L*. *donovani* and *L*. *infantum* [4]. Since the clinical features of VL mimic several other common diseases, accurate and early diagnosis is crucial for treatment and control of VL as the drugs currently used for chemotherapy have significant toxic side effects [5, 6].

Parasitological detection remains the gold standard for diagnosis of VL because of its high specificity [7]. However, as for all microscopic procedures, parasitological VL diagnosis is affected by variability in detection sensitivity (e.g. the sensitivity of bone marrow smears varies between 60% to 85% while that of splenic aspirates can exceed 95% [7]) and by the expertise of the microscopist. In addition, invasive bone marrow and spleen aspiration are painful and risky techniques. Culturing the parasite can improve the sensitivity of VL diagnosis but can be affected by contamination of bacteria or yeast species and are time-consuming [7].

Since a strong humoral response is generally induced in VL patients, serodiagnosis is an alternative to detection of the parasite in tissue samples. Serological tests for diagnosis of VL (e.g. enzyme-linked immunosorbent assay (ELISA), indirect fluorescence antibody test (IFAT), direct agglutination test (DAT) and immunochromatographic test (ICT)) are usually based on unpurified or recombinant antigens and can achieve sensitivities of >90% [8–11]. However, these tests cannot diagnose relapses as patients remain positive for several months or years after recovery [12, 13]. In addition, these test are limited in HIV patients co-infected with *Leishmania* where antibody response is very poor [14]. Molecular techniques such as polymerase chain reaction (PCR) assays have improved sensitivity and accuracy compared to parasitological and serological methods in the diagnosis of VL [15–17]. However, molecular techniques require competent technical personnel, sensitive equipment and continuous electricity supply, and are considerably more expensive than serological tests. Hence, molecular diagnostic tests are not suitable for the detection of VL in endemic regions under field conditions.

The detection of circulating pathogen antigens is an alternative immunodiagnostic test to identify an infection. This method is usually more specific than the detection of antibodies. In addition, antigen levels generally correlate with the pathogen load. In contrast to the detection of antibodies, antigen detection can be used to determine the treatment efficacy and to diagnose immunocompromised patients. The detection of circulating antigens can be performed as a lateral flow assay in the form of an ICT. This technique is a simple, rapid, and reliable method which can be easily carried out by inexperienced personnel under field condition. In this study we developed an ICT for the diagnosis of VL using monoclonal antibodies (mAbs) specific to an antigen of viscerotropic *Leishmania* species and compared the test with a commercially available ICT for the detection of anti-*L*. *donovani* antibody.

## Methods

### Ethics statement

This study was reviewed and approved by the Ethics Review Committee of the National Institute of Parasitic Diseases, Chinese Center for Disease Control and Prevention in Shanghai. All subjects gave their informed written consent specifying that their serum samples can be used for future studies. All serum samples were given a unique identification number to ensure that they were anonymized for any subsequent study.

Animal care and handling was in accordance with the standards specified in the Guidelines for the Care and Use of Laboratory Animals and approved by the Ethics Committee for Animal Care and Experimentation (SYXK2011-0127) and international animal experimentation guidelines were followed. The study and its protocols were approved by the Ethics Committee of the National Institute of Parasitic Diseases, Chinese Center for Disease Control and Prevention. All surgeries were performed under sodium pentobarbital anesthesia and every effort was made to minimize the suffering of the animals.

### Serum samples

A total of 213 serum samples from VL patients were investigated in this study: 135 serum samples were kindly provided by the Center for Disease Control and Prevention of Xinjiang Uygur Autonomous Region of which 64 were from an endemic area of anthroponotic type of VL (AVL) and 71 from an endemic area of desert sub-type of zoonotic VL (DST-ZVL), and 78 were kindly provided by the Gansu Provincial Center for Disease Control and Prevention which were from an endemic area of mountain sub-type of zoonotic VL (MST-ZVL). All VL patients were diagnosed by microscopic examinations of bone marrow smears.

In addition, 156 serum samples from patients with other diseases, including 31 with leprosy, 43 with malaria, 30 with cystic echinococcosis, 25 with schistosomiasis (*Schistosoma japonicum*), and 27 with toxoplasmosis as well as 78 serum samples from healthy donors were included as negative controls.

### Parasite culture and antigen preparation

Seven different *Leishmania* species and strains were used in this study (Table 1). The parasites were grown as promastigotes in 199 medium at 24°C. Promastigotes were harvested at logarithmic growth phase by centrifugation at 3000 × g for 15 min, washed 3 times with phosphate buffered saline (PBS) and lysed by re-suspension in approximately equal volume of deionized water containing 1 mM EDTA, 1 mM ε-aminocaproic acid and 1 mM dithiothreitol for 10 min on ice. Then, the cell suspension was subjected to three freeze-thaw cycles comprising liquid nitrogen to a 37°C warmed-up water bath, followed by three cycles of 1-min sonication. The lysates were centrifuged at 10000 × g for 30 min at 4°C. The supernatant was recovered and the protein concentration was determined using DC Protein Assay Kit (Bio-Rad Laboratories). The extract was stored at -20°C until use.

**Table 1:** *Leishmania* species used in this study.

Species	WHO code	Location of isolation	Host	Clinical form
*L*. *donovani*	MHOM/CN/80/XJ801	China (Xinjiang)	human	VL
*L*. *infantum*	MCAN/CN/90/SC	China (Sichuan)	dog	VL
*L*. *infantum*	MHOM/CN/08/JIASHI-1	China (Xinjiang)	human	VL
*L*. *aethiopica*	MHOM/ET/72/L100	Ethiopia (Wollo)	human	CL
*L*. *major*	MHOM/SU/75/5ASKH	Turkmenistan	human	CL
*L*. *tropica*	MHOM/SU/73/K27	Azerbaijan	human	CL
*L*. *braziliensis*	MHOM/BR/75/M2903	Brazil (State of Pará)	human	CL

### Production of monoclonal antibodies

Eight-week-old female BALB/c mice were immunized intraperitoneally with 50 μg soluble *L*. *donovani* MHOM/CN/80/XJ801 antigen emulsified in Freund's complete adjuvant. Four and eight weeks later, the mice received a booster intraperitoneal injection with the same amount of the antigen emulsified in Freund's incomplete adjuvant. Two weeks after the last injection, mice were tail-bled and the reactivity of sera was tested for the presence of anti-leishmanial antibodies by ELISA. Finally, mice were given an injection of 50 μg antigen through the caudal vein and three days later, the animals were killed and their spleens taken for generation of hybridomas.

Cell fusion and selection of hybridomas were carried out essentially as described by Köhler and Milstein [18]. Briefly, spleen lymphocytes from immunized mice were fused with SP2/0 murine myeloma cells at a 5:1 ratio using PEG 1000 (50%) as fusing agent. Hybridomas were selected and maintained as described by Malavasi et al. [19]. Culture supernatants were screened for the presence of antibodies by ELISA using promastigote extract as antigen. Antibody producing hybridomas were selected and cloned three times by limiting dilution. Ascites were obtained by injecting hybridomas into the peritoneal cavity of pristane-treated BALB/c mice (app. 5 × l0^6^ hybridomas per mouse). mAbs were purified from clarified ascites by ammonium sulfate precipitation and G protein chromatography. Class and subclass of mAbs were determined by ELISA using a Bio-Rad isotyping kit. Antibody titers during immunization and purification processes were determined by ELISA.

### Characterization of mAbs

The specificity of mAbs was determined by immunoblotting using soluble crude extract from the seven *Leishmania* species/strains as antigen (Table 1). The soluble crude antigens were separated by SDS-PAGE using a 10% polyacrylamide gel. Proteins were then electrophoretically transferred to nitrocellulose membranes which were blocked overnight with 5% skimmed milk in PBS-T (PBS containing 0.05% Tween 20) at room temperature. The membranes were washed once with PBS-T and then incubated overnight with the purified mAbs diluted 1: 20000 in PBS-T containing 5% skimmed milk at 4°C with constant shaking. Thereafter, membranes were washed three times with PBS-T for 20 min and then incubated with goat anti-mouse IgG secondary antibody conjugated to peroxidase diluted 1: 10000 in PBS containing 10% skimmed milk for 2 h at room temperature. The membranes were washed three times with PBS-T, once with PBS and developed with 0.05% 3,3-diaminobenzidine and 0.015% H_2_O_2_ dissolved in PBS for 2 to 3 min. With the onset of color development, the reaction was stopped by rinsing the membranes several times with double-distilled water.

### Preparation and application of ICT

Colloidal gold was prepared using the sodium citrate reduction method and mAbs were conjugated with colloidal gold following the method described by Roe et al. [20]. The mAb A_6_A_2_ and goat anti-mouse IgG antibody were sprayed onto nitrocellulose membranes in a 1-mm-wide line (0.8 μg/cm) as test and control line, respectively. Detecting reagent (mAb E_3_C_3_-colloidal gold conjugate) was incorporated into the sample pad. The test was carried out as follows: 10 μl of serum or 20 μl of whole blood (in 15 U/ml of heparin) was applied to the sample pad of the strip, then three drops of PBS were added, and the result was read after 15 min. The samples were judged as positive once a pink color appeared in the test line regardless of its intensity.

### Kalazar Detect Rapid Test assay

For comparison, the commercially available Kalazar Detect Rapid Test for visceral leishmaniasis (InBios International, Inc., Seattle, USA) was used. This diagnostic tool is an ICT for the detection of anti-*L*. *donovani* antibodies (anti-k39 antibody) in human serum and was carried out according to the manufacturer’s instructions.

### Data analysis

The definitions for sensitivity, specificity, true-positive values, false-positive values, true-negative values, false-negative values, and diagnostic efficiency were those as described by González-Sapienza et al. [21]. Sensitivity, specificity, true-positive values, false-positive values, true negative values and false-negative values were calculated from a 2×2 table. Chi-squared test was used for comparing the diagnostic parameters of the different tests. A *p* value of less than 0.05 was considered to be statistically significant. Kappa analysis was used to estimate the degrees of agreement between the newly developed ICT and the Kalazar Detect Rapid Test. A κ value of 0.81–1.00 indicates “almost perfect agreement”.

## Results

### Production of hybridomas and characterization of mAbs

After fusion of splenocytes isolated from a mouse immunized with soluble crude antigen of *L*. *donovani* MHOM/CN/80/XJ801 with cells of the murine myeloma line SP2/0, 12 hybridoma lines secreting specific mAbs were obtained following three rounds of cloning. The mAbs belonged to the IgG subclass IgG_1_ or IgG_2a_. When injecting the hybridomas in mice, the corresponding antibody titers of ascites produced by the animals were between 1:25600 and 1:204800.

**Fig 1:**
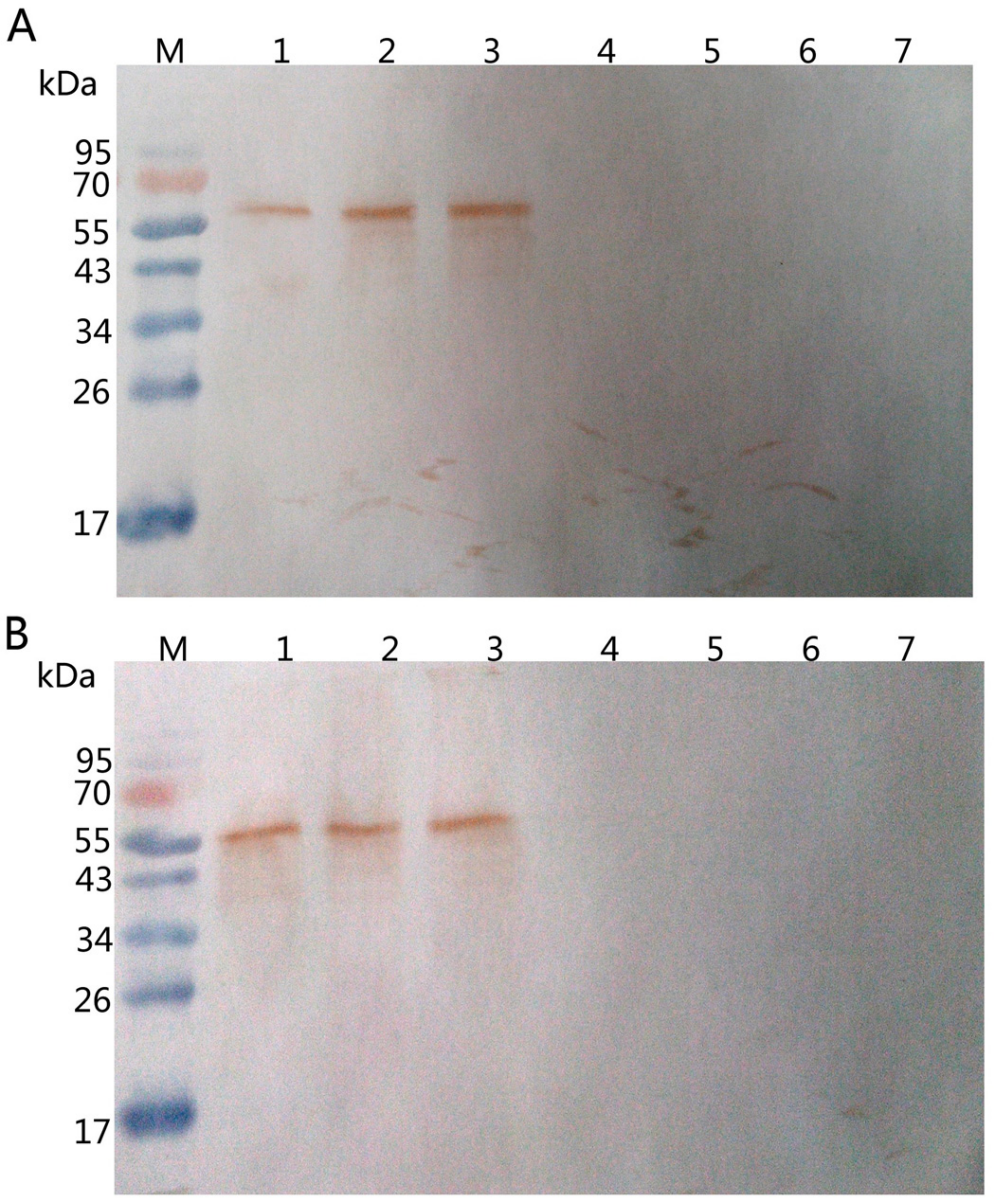
Western blot analysis of mAbs A_6_A_2_ and E_3_C_3_. Soluble crude antigen preparations form different *Leishmania* species/strains were analyzed by immunoblotting using mAbs A_6_A_2_ (**A**) and E_3_C_3_ (**B**). Lane M, molecular marker; lane 1, *L*. *donovani* MHOM/CN/80/XJ801; lane 2, *L*. *infantum* MCAN/CN/90/SC; lane 3, *L*. *infantum* MHOM/CN/08/JIASHI-1; lane 4, *L*. *aethiopica* MHOM/ET/72/L100; lane 5, *L*. *major* MHOM/SU/75/5ASKH; lane 6, *L*. *tropica* MHOM/SU/73/K27; lane 7, *L*. *braziliensis* MHOM/BR/75/M2903.

Further tests indicated that two mAbs labelled A_6_A_2_ and E_3_C_3_ may recognize the same antigen as determined by immunoblotting when using soluble crude antigen preparations from the seven different *Leishmania* species/strains (Fig 1). The analysis showed that both mAbs recognized an antigen of about 65 kDa from viscerotropic but not from dermotrophic *Leishmania* species/strains. Additional analysis indicated that both mAbs recognized the same epitope of the antigen (S1 Fig). Whether A_6_A_2_ and E_3_C_3_ are exactly the same mAbs remains to be determined. Nevertheless, the results suggested that the mAbs A_6_A_2_ and E_3_C_3_ may be suitable for the development of an ICT for the diagnosis of VL.

### Production of ICT strips

**Fig 2:**
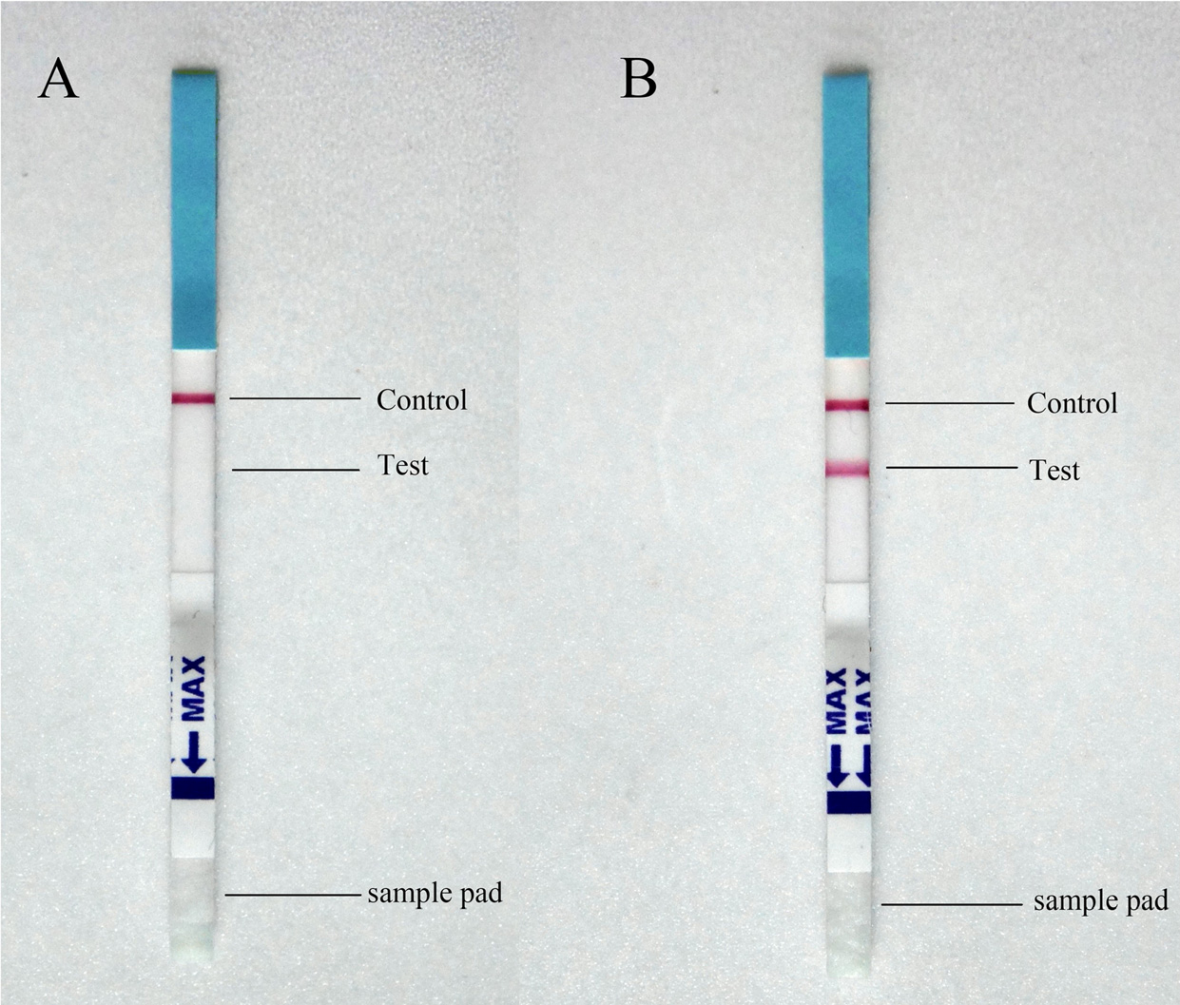
Examples of ICT strips tested with serum samples from patients. (**A**) Results with a negative serum sample: only the control line turned pink. (**B**) Results with a serum sample from a VL patient: both the control and the test line turned pink.

ICT strips to detect circulating *Leishmania* antigen were designed as a sandwich assay with immobilized A_6_A_2_ mAb as the capturing antibody (test line) and colloidal gold labelled E_3_C_3_ mAb as the detecting reagent. Immobilized goat anti-mouse IgG antibody was incorporated to confirm that the test operated correctly (control line). Fig 2 shows examples for the reactivity of ICT strips with two different serum samples. With a negative serum sample, only the control line developed (Fig 2A), while with a serum sample from a VL patient, both control and test lines turned pink (Fig 2B). This finding showed that the developed ICT was able to detect circulating *Leishmania* antigen in serum of VL patients.

### Evaluation of the ICT

The newly developed ICT was evaluated with 213 serum samples from VL patients. Of these, 95.8% (204) serum samples gave a positive reaction. With respect to the different endemic VL areas, 98.4% (63 out of 64), 93.0% (66 out of 71) and 96.2% (75 out of 78) serum samples from VL patients from AVL, DST-ZVL and MST-ZVL endemic regions showed a positive reaction, respectively (Table 2). The serum samples were also tested with the commercially available Kalazar Detect Rapid Test for VL, an ICT strip assay detecting antibodies of the *L*. *donovani* complex in human serum. Of the 213 serum samples, 94.8% (202) tested positive with the Kalazar Detect Rapid Test. The individual results for VL patients from AVL, DST-ZVL and MST-ZVL endemic areas were 100% (64 out of 64), 87.3% (62 out of 71) and 97.4% (76 out of 78), respectively (Table 2). Thus, the newly developed ICT detecting a circulating leishmanial antigen performed as well as the commercially available Kalazar Detect Rapid Test detecting an anti-leishmanial antibody.

**Table 2:** Number of positive serum samples from patients with VL and with other diseases, and from healthy individuals tested with the newly developed ICT and the Kalazar Detect Rapid Test.

Serum samples	No	No of positive samples	
		Newly developed ICT	Kalazar Detect
AVL	64	63	64
DST-ZVL	71	66	62
MST-ZVL	78	75	76
Leprosy	31	1	3
Malaria	43	1	1
Cystic echinococcosis	30	0	0
Schistosomiasis	25	0	0
Toxoplasmosis	27	1	1
Healthy donors	78	0	0

Next, both ICTs were evaluated with 156 serum samples from patient with other diseases. Only three and five serum samples gave a positive reaction with the newly developed ITC and the Kalazar Detect Rapid Test, respectively (Table 2). The tests were also checked with 78 serum samples from healthy donors. All control samples were found to be negative by both ICTs (Table 2).

In Table 3, the diagnostic parameters for the newly developed ICT and the Kalazar Detect Rapid Test for the detection of VL are presented. For all the diagnostic parameters, not many differences were found between the two ITCs. For both tests, sensitivity, specificity and diagnostic efficiency for the detection of VL were very similar being greater than 94% with similar 95% confidence intervals between 4–6 percentage points. Although statistical analysis revealed no significant differences between the diagnostic parameters (*p* > 0.4, χ^2^ < 0.5), the newly developed ICT performed about 1 percentage point better than the Kalazar Detect Rapid Test with respect to specificity, sensitivity and diagnostic efficiency. Apart from that, high degree of agreement was observed between the new and the commercial test for the detection of VL (κ = 0.986±0.008; 95% CI: 0.970 to 1.000).

**Table 3:** Diagnostic performance of the newly developed ICT and the Kalazar Detect Rapid Test in the detection of VL.

Diagnostic parameter	Newly developed ICT	Kalazar Detect
True positive (tp)	204	202
True negative (tn)	231	229
False positive (fp)	3	5
False negative (fn)	9	11
Sensitivity^a^	95.8% (95% CI: 92.1–98.1%)	94.8% (95% CI: 91.0–97.4%)
Specificity^b^	98.7% (95% CI: 96.3–99.7%)	97.9% (95% CI: 95.1–99.3%)
Diagnostic efficiency^c^	97.3% (95% CI: 95.4–98.6%)	96.4% (95% CI: 94.3–97.9%)

^a^ Sensitivity = tp × 100/(tp + fn)

^b^ Specificity = tn × 100/(tn + fp)

^c^ Diagnostic efficiency = (tn + tp) × 100/(tp + fp + tn + fn)

## Discussion

The diagnosis of VL presents many challenges because its clinical manifestations and symptoms are shared with other diseases such as malaria, disseminated tuberculosis, brucellosis, enteric fever, schistosomiasis and hematological malignancies [22, 23]. However, early detection in humans and dogs (as the main animal reservoir) infected with *Leishmania* is imperative if transmission of the disease is to be stopped in anthroponotic and zoonotic VL regions. Therefore, rapid and accurate diagnosis is an essential component of VL management and control. As viscerotropic *Leishmania* species usually induce a strong humoral immune response, serological tests detecting host antibodies offer a simple method for diagnosis of the disease. These tests have generally good sensitivity [7] but are unable to distinguish between a current and past infection due to the long persistence of parasite-specific antibodies in the body [8, 9]. In addition, serological methods may present the problem of cross-reactivity with antibodies from other infections (e.g. toxoplasmosis and malaria in the case of VL [24]) leading to false-positive results.

The limitations of serological methods can be overcome by antigen detection tests. Since the detection of *Leishmania* antigens would be indicative of the presence of the parasites in the body, these tests may be useful for screening programs, therapy monitoring and cure confirmation. A few antigen detection methods for the diagnosis of VL have been developed in recent times. Using Western blot technique, De Colmenares et al. reported the detection of two polypeptide fractions of 72–75 kDa and 123 kDa in the urine of VL patients while the antigens were not detectable in the urine of control individuals [25]. However, the test was only evaluated with 15 urine samples from VL patients and its sensitivity varied between 67% (for the 123 kDa fraction) and 93% (for the 72–75 kDa fraction). A heat-stable carbohydrate antigen detectable in the urine of VL patients was described by Attar et al. [26, 27]. Based on this antigen, a latex agglutination test was developed and when evaluated with urine samples from VL patients from Brazil, Yemen and Nepal showed an overall sensitivity of 81.4% [26]. However, the sensitivity of the test was disappointingly low (48–57%) in two field studies with confirmed VL patients from an endemic kala-azar region in Nepal [28]. By contrast, our newly developed ICT detecting a circulating antigen in blood or serum samples of VL patients performed much better (95.8% sensitivity). This is probably due to the fact that our ICT is based on a sandwich assay using two mAbs recognizing the same epitope that apparently occurs repeatedly on the antigen. It seems that mAbs are better diagnostic reagents as they recognize a single epitope with high specificity whereas polyclonal antibodies may be more sensitive but are often less specific.

The performance of our antigen detecting ICT was better than most antibody detection methods previously developed for diagnosis of VL. For example, various studies evaluating different immunological assays including ELISA, IFAT and DAT have shown that the sensitivities and specificities of these tests vary between 55–100% and 72–100%, respectively [7]. The performance of our ICT was also better than that of five other ICTs based on the rK39 antigen (rK39 ICT) which showed an overall sensitivity of 91.9% and an overall specificity of 92.5% [29]. The sensitivity of the rK39 ICT was lower in East Africa (85.3%) than in the Indian subcontinent (97.0%) [29]. These differences in sensitivity may be due to differences in antibody response in different ethnic groups and/or to differences in the K39 epitope in different strains of *Leishmania* species of the *L*. *donovani* complex. Whether our ICT will also perform equally well in other VL endemic regions outside of China remains to be shown.

Another advantage of our ICT over serological tests is that it is detecting a circulating parasite antigen and thus should be able to distinguish between re-infection and relapse in the case of recurrence, and should also be useful in diagnosing the disease in immunocompromised patients (e.g. HIV patients co-infected with VL). The results obtained with serum samples from VL patients from the DST-ZVL endemic area indicated that our ICT was indeed able to detect the disease in patients with a weak immune system. Most patients of this region were infants under one year old and thus at an age where their immune system is still underdeveloped producing only low antibody titers. Compared with the antibody detecting Kalazar Detect Rapid Test, our ITC detected 5.6% more cases of VL in this cohort (Table 2).

In the absence of a practical gold standard (the best gold standard in VL diagnosis is the culture of parasites from splenic aspirates), diagnostic tests are needed that can rapidly and accurately detect cases of VL under both field and laboratory conditions. The required performance and operational characteristics of a diagnostic test for VL case detection are ≥95% sensitivity, ≥98% specificity and ≤30 min time to result [23]. With a sensitivity of 95.8% (95% CI: 92.1% to 98.1%) and a specificity of 98.7% (95% CI: 96.3% to 99.7%), and with a development time of 15 min, our ICT fulfils these requirements. However, before our ICT can be recommended for clinical use, it needs to be validated in large-scale prospective studies outside of China in order to confirm its performance (sensitivity, specificity and reproducibility) and operational characteristics (user-friendliness and stability) in other VL endemic regions such as East Africa and South America.

## Supporting Information

S1 Fig mAbs A_6_A_2_ and E_3_C_3_ recognize the same epitope. Soluble crude antigen from *L*. *donovani* MHOM/CN/80/XJ801 (A and B) or buffer (C) were spotted onto nitrocellulose membranes. The membranes were block with PBS-T containing 5% skimmed milk powder and then washed with PBS-T. Membrane A was incubated with mAb E_3_C_3_-colloid gold conjugate (1:1000 diluted) in the presence of 100-fold excess of mAb A_6_A_2_ (1:10 diluted). Membranes B (positive control) and C (negative control) were incubated only with mAb E_3_C_3_-colloid gold conjugate (1:1000 diluted). After washing with PBS-T, no pink staining was observed for membrane A indicating that mAb A_6_A_2_ competed with mAb E_3_C_3_ for antigen binding. This result showed that both mAbs recognize the same epitope on the antigen.
(TIF)

S1 Checklist STARD checklist. (DOC)

S1 Flowchart Flow diagram for testing serum samples from VL patients by ICT. (DOC)
