# Supplementary figures and images for: Development of an Immunochromatographic Test for Diagnosis of Visceral Leishmaniasis Based on Detection of a Circulating Antigen
Source: PLoS Negl Trop Dis. 2015 Jun 30;9(6):e0003902. doi: 10.1371/journal.pntd.0003902 (PMC4488388; doi:10.1371/journal.pntd.0003902)

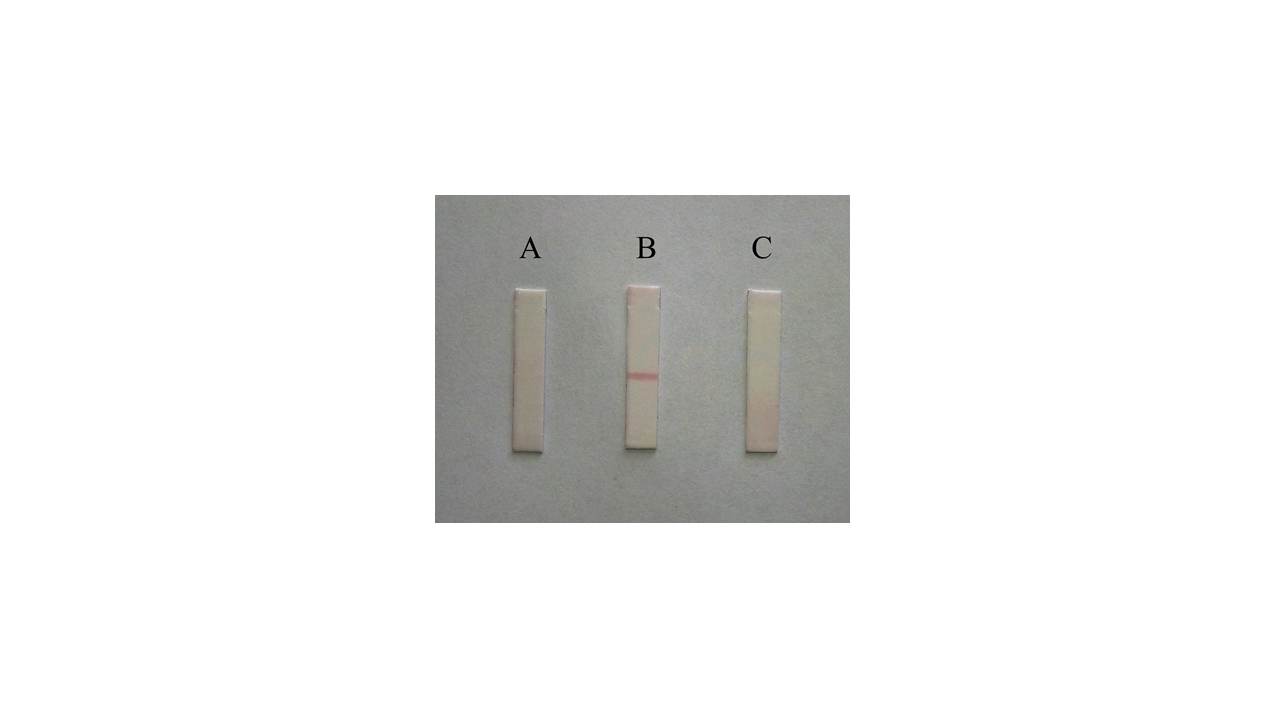

Supplement: S1 Fig — Soluble crude antigen from L. donovani MHOM/CN/80/XJ801 (A and B) or buffer (C) were spotted onto nitrocellulose membranes. The membranes were block with PBS-T containing 5% skimmed milk powder and then washed with PBS-T. Membrane A was incubated with mAb E3C3-colloid gold conjugate (1:1000 diluted) in the presence of 100-fold excess of mAb A6A2 (1:10 diluted). Membranes B (positive control) and C (negative control) were incubated only with mAb E3C3-colloid gold conjugate (1:1000 diluted). After washing with PBS-T, no pink staining was observed for membrane A indicating that mAb A6A2 competed with mAb E3C3 for antigen binding. This result showed that both mAbs recognize the same epitope on the antigen. (TIF) [file pntd.0003902.s001.tif]
