# Supplementary material for: Development of an Immunochromatographic Test for Diagnosis of Visceral Leishmaniasis Based on Detection of a Circulating Antigen
Source: PLoS Negl Trop Dis. 2015 Jun 30;9(6):e0003902. doi: 10.1371/journal.pntd.0003902 (PMC4488388; doi:10.1371/journal.pntd.0003902)
Supplement: Supplementary file 3 [file pntd.0003902.s003.doc]

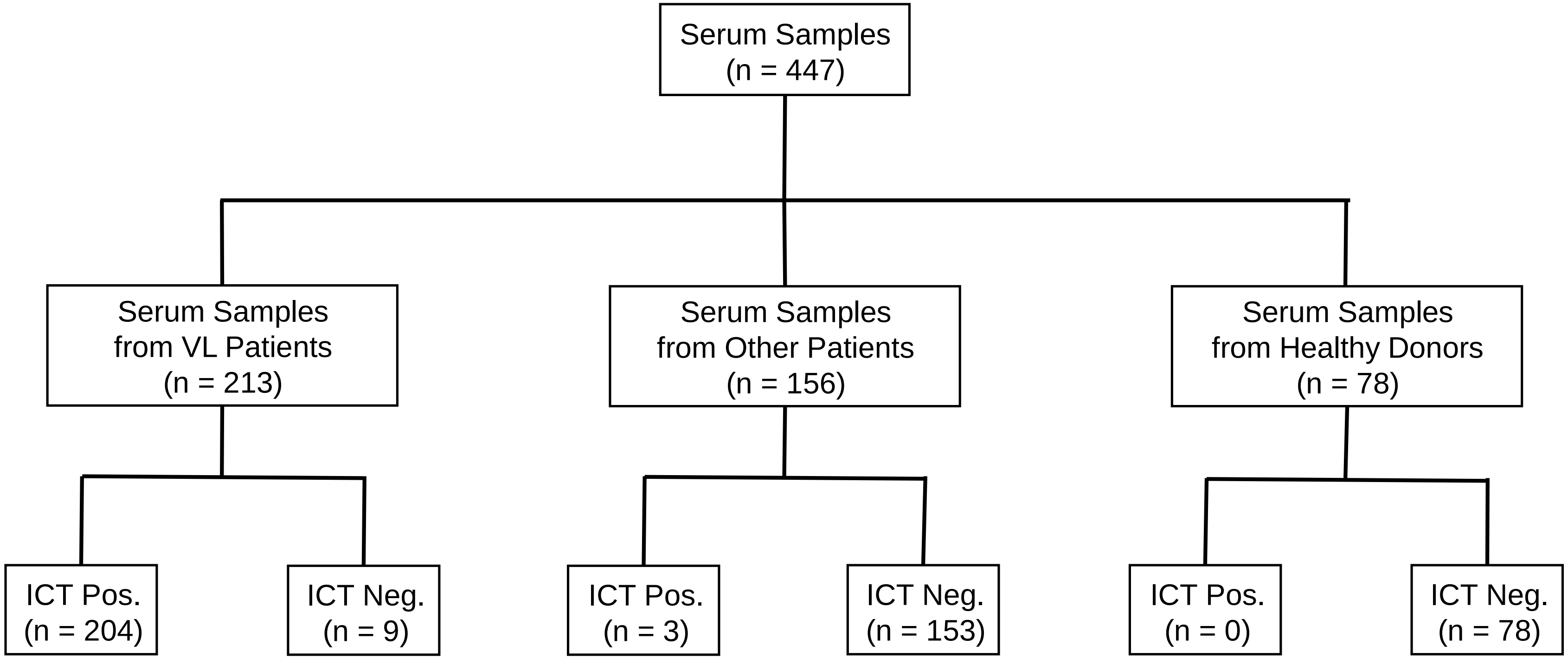
Serum Samples
(n = 447)
Serum Samples
from VL Patients
(n = 213)
Serum Samples
from Other Patients
(n = 156)
Serum Samples
from Healthy Donors
(n = 78)
ICT Pos.
(n = 204)
ICT Neg.
(n = 9)
ICT Pos.
(n = 3)
ICT Neg.
(n = 153)
ICT Pos.
(n = 0)
ICT Neg.
(n = 78)
